# The importance of terminology, lived experience inclusion and scientific discussion regarding end-of-life care in anorexia nervosa: a response to Gaudiani et al.

**DOI:** 10.1186/s40337-023-00872-2

**Published:** 2023-08-25

**Authors:** Andrea Phillipou

**Affiliations:** 1 Orygen, 35 Poplar Rd, Parkville, Melbourne, VIC 3052 Australia; 2 Centre for Youth Mental Health, The University of Melbourne, Melbourne, Australia; 3 Department of Mental Health, St Vincent’s Hospital, Melbourne, Australia; 4 Department of Mental Health, Austin Health, Melbourne, Australia; 5 Department of Psychological Sciences, Swinburne University of Technology, Melbourne, Australia

**Keywords:** Anorexia nervosa, Eating disorder, Terminal, End-of-life care, Criteria, Definition, Lived experience

## Abstract

Whether or not to define ‘terminal anorexia nervosa’ has been a hotly debated topic in the eating disorders field recently. Being able to have open scientific debate on important topics such as this is essential for the progress of our field—but needs to be undertaken respectfully, allowing all perspectives to be heard. My personal perspective on this topic comes from being a researcher who sees individuals with anorexia nervosa (AN) across all stages of illness and recovery, as well as having had a loved one die from AN. Although I disagree with the terminology of ‘terminal AN’ and believe that establishing criteria has the potential to cause harm, I strongly believe in showing compassion to individuals with AN across all illness stages, including those who may wish to seek end-of-life care. This is a complex issue that our field requires guidance on, and we need to work in genuine collaboration with individuals with lived experience of AN to figure out how to appropriately approach end-of-life care when it is warranted.

Anorexia nervosa (AN) can be terminal—and is in many cases. The Gaudiani et al. [1] paper proposing that we should consider if and how we should define ‘terminal AN’ has led to important discussions within our field, particularly giving individuals with lived experience an important platform to share their thoughts and concerns on this sensitive topic (e.g. [2–5]). Being a researcher without personal lived experience of AN, I have been reluctant to weigh in on the discussion as I would not be in the unenviable position of either a clinician or an individual with AN having to make decisions on ceasing treatment and pursuing end-of-life care if ‘terminal AN’ criteria were established. I am, however, in the unfortunate position of having lost a loved one to AN; my aunty died from the physical complications of AN in 2008, aged 35, after a long and severe course of the illness. Being open about my family history in my professional life means that since the Gaudiani et al. [1] paper was first published, I have been asked on countless occasions where I stand on the issue of defining ‘terminal AN’. As someone who tends to be vocal about various issues in our field (and likes to stir the pot to encourage scientific debate; see [6–9]), I find myself unable to form a strong opinion favouring either side of this argument. What I do feel strongly about though, is that we need to be having these discussions—and we need to do so in a respectful and considered way. This is a complex and sensitive issue that requires consideration from all perspectives, even when those perspectives do not alight with our own. In terms of my own opinion on the matter—through the lens of a researcher who sees individuals with AN across illness severities and across the spectrum of recovery, as well as having watched one of my favourite people in the world suffer a long and painful illness and death from AN—I can see both sides of the argument and my perspective falls somewhere in between.

While I believe that we need to be able to have open discussions regarding whether we define end-of-life care for AN, the terminology used to refer to ‘terminal AN’ is problematic. Unlike other medical illnesses which can directly lead to death, illness course is much more complex and varied for mental illnesses and are not terminal in the same sense. The feedback from individuals with lived experience has also been loud and clear that this terminology is not appropriate, and the concept of ‘terminal AN’ has the potential to cause harm [2–5]. These authors have provided strong and well-reasoned arguments identifying the many issues that would result if ‘terminal AN’ criteria were determined—which I wholeheartedly agree with—including submitting to the ambivalence that is often present in terms of treatment and recovery, losing hope that recovery is possible, and that they are not deserving of treatment. These perspectives are of particular importance as they not only come from people with lived experience of AN, but also from several individuals who would have met the ‘terminal AN’ criteria proposed by Gaudiani et al. [1] but were able to re-engage in treatment. This highlights the critical importance of incorporating the voice of lived experience in the work we do, and if end-of-life care criteria or guidelines are to be pursued, that they are co-developed with a diverse range of individuals with lived experience of AN including those with severe and long-standing illness who have re-engaged in treatment, as well as those who have not.

Despite disagreeing with the terminology used by Gaudiani et al. [1] regarding ‘terminal AN’, I do agree with the authors that we need guidance on end-of-life care in this space. The reality is, that despite best efforts, our treatments are not effective for many with AN, and several will sadly either die from the physical consequences of the illness or will take their own lives. While recovery is possible after even very long-standing illness [10], our field is nowhere near at the stage where we are able to identify who will and who will not respond to treatments—which makes establishing criteria for determining someone with ‘terminal AN’ virtually impossible. Internationally, there are efforts being made to improve treatments and consequent outcomes for individuals with AN. While I am hopeful that new and more effective treatments will be developed, research is slow-moving and we need to concurrently support those currently in need of end-of-life care. While it is imperative that we continue to provide hope to individuals with AN that they can recover despite the severity and length of their illness, we do also need to acknowledge that there are many who will die from the illness, and we need guidance on how to compassionately approach end-of-life care for these individuals. Defining criteria, however, is problematic given the rigid nature of employing categorical approaches. As such, it may be more appropriate to establish guidelines that provide (non-binding) recommendations and guidance. The establishment of any such guidelines should be undertaken collaboratively with diverse voices of those with lived experience, and if they are to be employed, to be administered cautiously on a case-by-case basis, and in deep consultation with the individual and their family. While we should always endeavour to pursue treatment and recovery, we also need to show compassion for those at every illness stage. Rarely discussed in the AN space is the concept of dignity of risk—i.e. giving individuals the right to make decisions for themselves, even when there may be concerns about their capacity to do so. Dignity of risk is applied widely throughout the aged care and disability sector, and while decision-making capacity may be considered to be reduced in some of these individuals, affording people the freedom to make decisions about their own lives is paramount. This concept challenges our very instinct to protect others from harm but may be a more compassionate approach, particularly if we can ensure that the individual is expressing their true wishes rather than their decisions being driven specifically by their AN. The traditional concept of dignity of risk involves taking ‘reasonable risks’ to improve dignity and quality of life, and it is important for us to understand how this concept may be extended to end-of-life care in AN and, critically, whether the concept resonates with individuals with AN or not.

Defining ‘terminal AN’—or more appropriately, end-of-life care—is an important topic that requires considerate, thoughtful and respectful discussion. I am thankful to Gaudiani et al. [1] for initiating the dialogue on this vital issue. Ultimately, Gaudiani et al. [1] argue for compassionate care for those who wish to end their life. While I have concerns with the terminology used and the proposal to establish criteria, I agree with the authors that clarity is needed on how to determine if end-of-life care is warranted and how to approach it if so. It is essential that if any guidelines on end-of-life care for AN are to be produced, that they are co-designed with those with lived experience to ensure that hope can still be maintained that recovery is possible irrespective of length or severity of illness; in addition to ensuring the voice of lived experience determines the content of any such guidelines. By taking a thoughtful, considerate and compassionate approach to end-of-life care, it does not mean that we need to lose hope, but that all individuals with AN are treated with the dignity and respect that they deserve. Despite my aunty being so unwell for such a long time, I don’t believe she would have pursued end-of-life care if it was offered to her—but I would have wanted her to be given the dignity to make that decision for herself.

## Data Availability

Not applicable.

## Appendix

Addendum

*Review of "The importance of terminology, lived experience inclusion and scientific discussion regarding end-of-life care in anorexia nervosa: A response to Gaudiani, *et al*." by Dr June Alexander.*

My perspectives on this topic come from more than four decades of personal experience with anorexia nervosa, plus as a researcher, and as a mentor for the past 15 years with people who are living with severe and enduring anorexia nervosa.

Like Andrea, I disagree with the label ‘terminal AN’ because this can be seen as a validation, an ultimate goal, for people with this challenging illness. The illness seeks to isolate and destroy, and would be strongly attracted to, and aligned with, the word ‘terminal’.

At the same time, I concur with Andrea that compassion towards individuals with AN is vital across all stages, including those who want to end their life.

My experience has led me to hold the view that there is hope at every age. I prefer to see a focus on hope, rather than creating a label that validates death as an outcome.

AN can be terminal. Many people with the illness do die. However, I’ve yet to meet a person who has clawed their way back from the precipice of death and regrets that they are alive today. I was one of these people. Forty-plus years is a long time to struggle with an illness in the mind. I am ever grateful to the doctors did NOT give up on me. I am a grandmother now. My grandchildren call me, ‘Grandma’ and every day I give thanks that I am here to hear their voices and pamper them with love.

I am ever grateful to the psychiatrist who patiently encouraged me for more than 30 years, in finding a pathway to reconnect with my healthy self. I credit him with saving my life. He could have given up; he could have labelled me non-compliant; he could have told me to go home and die; when I was suicidal, he believed in the ‘me’ I could not see, he always treated me with respect, and eventually I gained the courage to believe and trust in him, more than in my AN. My recovery of healthy self after decades of struggle was described as a miracle.

Give up? NO!

Definitely, I agree with Andrea that the term ‘terminal AN’ is problematic.

Anorexia, especially, is a complex illness. There are similarities in each case, but also there are differences. Rarely is anorexia a single diagnosis. Co-morbidities are common. Treating the co-morbidities (e.g., in my case, chronic anxiety and depression, trauma) can assist in easing the effects of the anorexia. To apply the label of ‘terminal AN’ would feed the illness, not assist recovery.

Andrea points out that ambivalence is a common theme in terms of treatment and recovery—and this is why it is very important that the treatment team members don’t give up on their patient—they need to keep their door open—give the patient a thread of hope even when all seems lost.

Yes, there will be deaths—from organ or other physical failure, from suicide—there is only so much a body can take—but to ‘predict’ a termination of a life wracked with AN by placing a label on suspect patients would be fraught with dangerous risk of misinterpretation.

Hope must be the lantern that lights our path.

There were multiple times when I would have chosen death if offered as a way out of my struggle. The worst outcome for me would have been if my psychiatrist had joined others (when we met, I had experienced six desperate years of misdiagnosis) and said, ‘Sorry, I can’t help you—you have had the illness too long to expect any change,’ I would not be writing this response to Andrea’s letter. I would not have seen my children grow up and marry. I would not have seen my grandchildren. I would have been a suicide statistic.

That said, I agree that ‘recovery’ is not possible for everyone. I find the word ‘recovery’ is not always appropriate for someone who has reconnected with their healthy self after decades with anorexia. Those decades with anorexia do not miraculously disappear. They remain part of one’s life experience. We can never be the person we would have been if we had not developed the illness. So, we cannot ‘recover’ who we were prior to the illness. There is a great canyon of life experience to catch up on. I prefer the term, ‘ongoing healing’.

I say this because many people with SEAN do not want to ‘recover’. They feel safe within their illness. Rather than pressuring and expecting them to ‘recover’ (and making them feel hopeless and suicidal when they fail) it is far better to focus on ‘improving life quality’, and with this focus, amazing progress can be achieved. Tiny steps forward can lead to bigger steps later. For instance, rather than insisting on all meals and snacks, focus on arranging an out with a support worker, going to a café for a coffee, or sitting by the sea, feeling the breeze on one’s face and the sand on one’s feet. Such experiences can help remind the patient of the beautiful life that exists beyond their illness and strengthen their will to live. As Andrea notes, it is important for the patient to be part of the treatment team; it is important for the patient, no matter what stage of the illness they are at, to be given a choice. It could be a choice between going to a café or going to see a movie; it could be between eating food or having an NGT. Or it could be, ‘Okay, you must have an Ensure—what flavor do you choose?’.

The opportunity to have even a small choice gives the patient a sense of being respected, of having some control over their life and the situation, and this is very important. Always LISTEN to the patient and treat them as a person who deserves to be treated with respect, not as an illness. Be compassionate, not punitive.

Personally, I don’t see any validity for ‘terminal AN’ labelling. If a patient is very ill because of their AN, they can be given palliative care—without the label—because, you never know, they may rally and claw their way back to a healthy life.

Do not deny them the opportunity to grab this thread of hope.

June Alexander.

## Abbreviation

AN: Anorexia nervosa
